# 76 A Retrospective Review of Factors Influencing Return to Work/school in Burn Patients

**DOI:** 10.1093/jbcr/irac012.079

**Published:** 2022-03-23

**Authors:** Kevin N Foster, Claudia Islas, Samin Kabir, Karen Richey

**Affiliations:** The Arizona Burn Center Valleywise Health, Phoenix, Arizona; Arizona Burn Center Valleywise Health, Phoenix, Arizona; Arizona Burn Center Valleywise Health, Phoenix, Arizona; Arizona Burn Center Valleywise Health, Phoenix, Arizona

## Abstract

**Introduction:**

Treatment and time to healing for burn injuries is largely dependent on the depth of the burn, location, and size. Treatment varies and can include any or several forms of dressings, surgery to remove eschar, allografting, autografting, depending on the depth of the burn. Hospitalization time ranges from days to months. Once discharged, patients continue to follow-up with burn specialists to ensure burns are healing properly. The time it takes a burn patient to return to work or school differ and can be dependent on many factors including personal choice. The objective of this study was to assess the time it took patients to return to work or school following burn injuries and help identify potential factors influencing their return time.

**Methods:**

A retrospective chart review of patients aged > 5 years, with TBSA > 10%, requiring hospitalization for > 7 days in a two-year span (2018-2019) was performed. Patients who did not report employment during admission and those who expired during hospitalization were excluded. IRB approval was obtained to contact patients via telephone who did not report a return to work or school date during their outpatient follow-up.

**Results:**

There were a total of 1579 burn admissions from 2018-2019, 93 of those patients met final protocol criteria. Seventy-four of those patients returned to work/school (RTW), and nineteen did not return to work/school (N-RTW) as of chart review date. The average total body surface area (% TBSA) for RTW was 18.30 vs. 33.08 for NRTW (p=0.0002). The average length of stay (LOS) for RTW was 23.55 vs. N-RTW 51.15 (p=0.0102). Exact return to work/school dates were obtained from 67 patients. The average length of days to return to work/school (n=67) was 102.19 post discharge, the minimum was 2 days, and the maximum was 785 days. There were 9 patients in the N-RTW group who filed for disability.

**Conclusions:**

Results suggest the average days per %TBSA it takes for burn patients to return to work or school is 5 days. Additionally, larger %TBSA burned and longer hospital LOS also adversely affected return to work. Additional studies are needed to identify additional factors influencing return to work and to identify methods to hopefully improve the ability to return to work.

